# Everyday Agency: Rethinking Refugee Women’s Agency in Specific Cultural Contexts

**DOI:** 10.3389/fpsyg.2021.726729

**Published:** 2021-11-17

**Authors:** Maria Kanal, Susan B. Rottmann

**Affiliations:** ^1^Institute of Religious Studies, Jagiellonian University, Kraków, Poland; ^2^Department of Humanities and Social Sciences, Faculty of Social Sciences, Özyeğin Üniversitesi, Istanbul, Turkey

**Keywords:** forced migration, refugee women, agency, coping, cultural coping

## Abstract

This article proposes an interdisciplinary approach to refugee agency – the capacity to act within structural conditions – using the example of Syrian women rebuilding family and home in Turkey. Our broader objective is to prompt a re-thinking of refugee women’s everyday agency for scholars researching migration. The dominant manner of studying agency tends to be centered on refugees’ efforts to change their particular situations. Drawing on the latest theoretical propositions of cultural psychology (collective coping and the cultural coping model), we argue that agency can also be observed through examining how refugees rebuild their lives in the face of the many changes and challenges they have experienced. Guided by the cultural coping model, we describe stressors and coping strategies in context. With this approach, we can escape the trap of viewing refugee women in dichotomous ways, either as traumatized victims or as liberated from “traditional patriarchy.” A total of 33 semi-structured interviews were conducted in Turkey with Syrian, Arabic-speaking adult women. Interviews aimed to obtain comprehensive narratives on acculturation, daily stressors, coping strategies and everyday experiences of uprootedness. We used constructivist grounded theory (Charmaz, 2006) to identify significant themes (initial coding) and then code for more conceptual units of meaning (focused coding). The findings are structured around context specific themes: stressors and coping strategies. The study revealed three important types of stressors: family-related, role-related and place-related stressors. Each stressor can only be understood within the cultural context of inter-dependent agency, motherhood and neighborhood belonging, which are highly valued lived experiences of the refugee women. The study also identified three coping strategies: faith-based, home-making and identity building strategies. Our research shows that relying on Islamic understandings, creating the routines of a happy home and forging neighborly ties are important gender and culture specific manifestations of agency. The value of this research is that it provides migration scholars a useful model for designing research with female refugees. By identifying and writing about these specific and contextual forms of agency, researchers can provide better support to refugee women in their daily lives, while also challenging the image of passive “womenandchildren.”

## Introduction

The purpose of this article is to broaden the default understanding of agency used in migration studies and by doing so to increase awareness of the everyday agency of refugee women. Despite notable exceptions (cf. Jahan, 2011; Fiddian-Qasmiyeh, 2016), female refugees are often seen as non-agentic, passive statistics. When they are seen as agents, their agency is often characterized as a strategy of an independent actor struggling against her culture. Feminists and postcolonial studies scholars have criticized conceptions of agency based on liberal, autonomous individual subjectivity, noting that such conceptions are Eurocentric and particularly problematic when applied to Muslim women (Mahmood, 2005). Yet, within migration studies, scholars have tended to maintain the focus on liberal personhood in order to show how migrants resist oppressive structures before and after migration (Paret and Gleeson, 2016). Drawing from research with Muslim refugee women in Turkey, we demonstrate a way of re-thinking refugee agency that goes beyond a focus on whether individuals “challenge” or “uphold” norms to look at how they make decisions and take actions within their cultural contexts. The value of this research is that it provides migration scholars a useful model for designing research with female refugees.

We argue that agency is only comprehensible within particular contexts (Kitayama and Uchida, 2005; Menon and Ho-Ying Fu, 2006). For the migrants in our case study, this context is one in which embedding in family roles and a sense of community belonging are two main goals. By embedding in context, our research combats portrayals of Syrian women as helpless victims. Women’s agency, similar to women’s work, becomes invisible if judged according to the wrong criteria. To highlight agency, we undertake an analysis of stressors in cultural context (family-related, role-related, and place-related) and cultural values surrounding family, work and religion. We also identify particular realms of action according to gender and cultural norms. We also go further by reflecting on agency via the concept of cultural coping developed in the discipline of psychology. A cultural coping model is useful for a few reasons. First, refugees usually have experienced significant rupture caused by migration and often are living with on-going hardship. Their daily lives and life goals are actually centered on coping with stressors and developing strategies related to this. Second, coping is interested in the ways that people adjust to circumstances as best as they can. An important question for researchers studying refugees is the mental health impact of being agentive (cf. Gigengack, 2008): is “agency” directed toward improving one’s personal circumstances by exerting one’s will on the world (Menon and Ho-Ying Fu, 2006) the only type of agency there is, and is it always something to be celebrated? We find that by acting *within* cultural constraints – without trying to directly oppose them – refugees create a variety of manifestations of agency.

We also argue that active change is not the only sense of agency that can be observed. Women can also be seen engaging in secondary control measures, involving carrying on with life, “going with the flow” or “trying to fit into the world,” and these should also be seen as forms of agency. Moreover, this is a healthy form of action. Trying to “stay where you are”’ and accommodate makes a lot of adaptive sense for people who have been uprooted. In fact, finding a place where a person belongs is a primary need of all of us and especially migrants (Baumeister and Leary, 1995; Morling and Evered, 2006). By focusing on how refugees practice useful coping strategies by placing experiences within Islamic religious understandings, focusing on daily life, routines and engaging in neighborly relations, we show that women demonstrate significant agency.

The paper begins with a discussion of how agency has been conceptualized within the social sciences, feminist research, migration studies and psychology, and we outline our main approach to analyzing agency in this article. Then, we describe the context of Syrian migration to Turkey and the research methods used in our study. Next, we present our findings via a close analysis of stressors and coping strategies. The paper concludes with a discussion of the influence of culture and gender on women’s stressors and a reflection of how coping is shaped by available cultural resources. Finally, the conclusion discusses the significance of this research for analyzing agency in other migration contexts.

## Theorizing Everyday Agency

Social and behavioral scientists have always wanted to understand the capacity of individuals to act within (and possibly change) their social groups and structures. Yet, the concept of agency is widely described as a slippery term, because its definitions are so diverse. As a result, the concept remains poorly defined. A significant contribution to theorizing agency which shaped the study of the topic for decades was sociologist Pierre Bourdieu’s (1977) “theory of practice.” Bourdieu characterized social life as a dialectic between structure and agency, and he helped to move sociology and related disciplines away from static conceptions of society and culture and to account for systemic dynamism. It was heralded as the most important new direction for anthropology (Ortner, 1984). Yet, Bourdieu himself and those who followed his approach tended to focus on how actors reproduce the societal system rather than their capacity for systemic change. In the following decades, scholars sought to further characterize the agency side of practice theory with concepts such as “structuration” (Giddens, 1984), “tactics” (de Certeau, 1984), “resistance” (Scott, 1976), and cultural “creativity” (Lavie et al., 1993).

Feminist and postcolonial studies scholars initially embraced the notion of agency because they wanted to understand how subordinated members of societies (e.g., women, migrants, ethnic, and racial minorities) could resist their subjugation. Recent feminist scholars criticized early theories of agency for being too binary (a “passive victim” was opposed to an “agent”), Eurocentric and primarily based on a Western liberal conception of autonomous individual personhood (Spivak, 1988; Mahmood, 2005). For example, Saba Mahmood studied Egyptian women in the Islamic revival movement and demonstrated that “the capacity to endure, suffer and persist is a form of agency”(Mahmood, 2001, p. 217). Her theories have been particularly important for helping us to understand the needs, goals and desires of Muslim women as agentic within structures of subordination. This is an approach that we draw from in this article. Another important contribution to changing the narrative on women’s agency comes from Judith Butler (2016) who provides a systematic criticism of the phenomenon she calls “projected vulnerability” which happens when people assume that a certain group is vulnerable. She criticized conceptions that make the inferential leap to assert that vulnerability is associated with passivity and victimization, or to put it in a more straightforward manner: “vulnerability excludes agency.” Vulnerability can be a resource for agency and by decoupling vulnerability and agency, feminist researchers can call for additional legal protection for women refugees as a vulnerable group while simultaneously engaging in efforts to dismantle harmful images of “victimized refugee women.”

Within migration studies, agency is usually understood in terms of migrants’ decision-making strategies about mobility (Bakewell, 2010; Feng et al., 2020). As men are usually perceived to be the primary decision-makers, the widespread representation of refugee women is “one of apolitical, non-agentic innocents in need of protection” (Fiddian-Qasmiyeh, 2016). Women’s agency is only perceived by researchers if it takes the form of opposing the oppression or exploitation they face from their own culture’s patriarchal structures or in host countries. Further, it is usually seen as a positive force of change (Paret and Gleeson, 2016). Agency is equated with disruption. Thus, agency is not seen as evident in acting as expected in daily life. Agency is rarely visible to researchers via the performance of approved social roles.

Psychologists conceptualize agency as “the capacity to perform actions that are in line with a person’s conscious goals and intentions” (Haggard and Eitam, 2015). Agency is seen as one of the core social motives (Wojciszke, 2018), closely related to effectiveness or sense of control (Bandura, 2006; Fiske, 2013) and is understood as personal efficacy as opposed to reliance on others. This basic understanding of agency, which is meant to be applicable to any human being regardless of their cultural context, is very often used with the default understanding of a “person” as an independent individual. As such, agency is seen as a form of control whereby one can influence the environment in accordance to one’s desires, needs and goals.

This individualist approach to agency has been criticized as ethnocentric (Menon and Ho-Ying Fu, 2006). Kitayama and Uchida (2005), rethinking the concept from the cultural psychology perspective, named this type of agency “independent agency,” common in individualistic cultures. They theorize it as a consequence of the distinction between independent and interdependent constructions of self and argue that people with an independent kind of agency set goals and take actions to fulfill their personal desires and needs. In the second kind of agency (interdependent), actions take the form of adjustment to the expectations of others. The self is seen as interdependent (defined in relationship to others), and the desires and goals of others are incorporated into personal goals. We find this distinction useful for theorizing refugee agency. Social psychology has also contributed to the debate on agency by distinguishing between actions related to the interests of the self, as opposed to communion which is related to the interests of others (Abele and Wojciszke, 2007). Communion contains such motives as focus on other people’s well-being and conformity. The most important insight from this way of thinking for the discussion of the agency of refugee women is recognition that any division between self-profitability and other-profitability in defining goals will be very much dependent on the nature of the relationship we have with the other person and our point of view (Abele and Wojciszke, 2007).

Some theories seek to move beyond this “wrangling dualism” of agency against communality or independence against interdependence. Bandura (2008) proposes three modes of agency that every person uses on a daily basis, regardless of the cultural setting: individual, proxy and collective. By individual agency, he means achieving goals by one’s own influence and resources; proxy agency is defined as using others with more control to achieve desired outcomes; while collective agency means setting a common goal and achieving it by “pooling the resources and skills of the group members” (Bandura, 2008). Bandura assures us that any individual, regardless of their cultural background, will always use all of the levels of agency to make it through the day, however the proportions may differ. Although Bandura’s theory was not developed to explain migrant agency in the cultural context, it can be a useful tool for migration researchers looking to broaden their understanding of agency.

In the forced migration context, a sense of self-efficacy is endangered by the loss of control over a refugee’s life situation. An important question here is one of the adaptive sense of acting against all odds. Active acceptance of one’s powerlessness in the context of actual powerlessness can be adaptive and lead to positive mental health outcomes (Nakamura and Orth, 2005; Park, 2010). But, this refusal to challenge one’s circumstances should not be seen as evidencing a lack of agency. Instead, it requires a different approach to agency.

**FIGURE 1:**
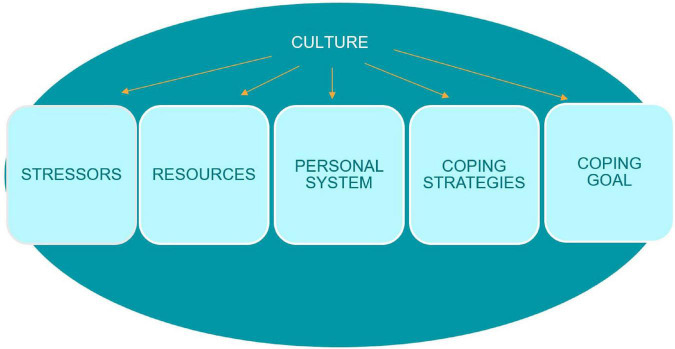
Culture’s influence on the coping model, adapted by Kuo (2013) based on Chun et al. (2006). Included courtesy of the author.

Our study draws on the above mentioned approaches to agency, but also introduces the cultural coping model (Chun et al., 2006) for the purpose of reflecting on refugee agency. We chose the following model (see Figure 1) to explain how forced migrants experience different stressors depending on the cultural context and assigns various levels of stressfulness to them. The model takes into account that different resources are available and perceived as useful and that personal systems will vary based on cultural contexts. Also, different coping strategies will be recommended and transmitted through generations. In order to analyze our findings, we focus on stressors and coping strategies, as these are the two dynamics that relate most directly to agency. What is crucial for understanding the connection between coping and agency is the recognition of different goals that are set and the outcomes that are wished for by the individual. The concept of collectivist coping is important here. Relying on the interdependent self, collective coping (Kuo, 2013) is other-oriented. It means that a person faced with a difficult situation includes other people’s needs while choosing a coping strategy or setting a coping goal, but also that the repertoire of preferred coping behaviors are rooted in the collectivistic values of a society (Yeh et al., 2006; Kuo, 2012).

## Context and Methodology

### Case Background

Since the outbreak of civil war in 2011, Syrians have been migrating to Turkey in large numbers, and there are now nearly 3.6 million in the country (Republic of Turkey Directorate General of Migration Management [DGMM], 2020). Most live in major Turkish cities with the highest population in Istanbul and in the country’s South East provinces (such as Hatay) (Attention Interest Desire Action [AIDA], 2019). They are not officially refugees because Turkey maintains a geographic limitation to the Geneva Convention that excludes non-European refugees (Cetin et al., 2018). Instead, they have Temporary Protection Status (TPS), which confers the right to remain in Turkey for an unlimited amount of time, but they must obtain a work permit if they wish to work, and there is no direct path to citizenship or political representation (Baban et al., 2016). Initially, Syrians were welcomed by Turkey’s political leaders who referred to them as religious brothers (*ansar*) and honored guests. However, the discourse has changed in recent months, with leaders suggesting that Syrians will be returning to their country very soon, despite an on-going war and a global pandemic. The local population is also increasingly hostile toward migrants with a recent survey reporting that 86% of Turks feel that Syrians should be sent home (Erdoğan, 2018). Half of all Syrian migrants are women (UN Women, 2018). There are several reports outlining gender-specific problems/stressors (i.e., UN Women, 2018; Ozden and Ramadan, 2019), however we lack research on agency and coping strategies.

### Research Design and Reflexivity

This article emerges from a collaboration between the two researchers who conducted their research between 2017 and 2019. We are a psychologist and an anthropologist, and this research embodies the three major epistemic values of interdisciplinarity, which are breadth, integration and transformation (Huutoniemi and Rafols, 2017). Breadth is achieved by studying the details of the coping process through the lens of intersectionality without restricting the study to the pre-established categories of any one discipline (e.g., religious coping questionnaires in psychology). Our research also involves the integration of knowledge from disparate fields (cultural psychology, medical anthropology, psychology of religion and gender studies) and aims to deliver a coherent intellectual synthesis of the findings, understandable for academics from a variety of disciplines working with forced migrants. By recognizing both the social and also the cognitive dimension of academic narratives on migration, it contributes to a transformation in the way academia and the general public perceive forced migrant women.

We are both women, which facilitated access to and comfort for the women participants who are the focus of the study. For cultural reasons and because of the matters discussed (religion, personal experiences, and motherhood), most participants would not have been willing to meet with a male researcher or to speak openly. Many encounters happened in participants’ homes, which fostered an intimate environment and again, would have been impossible for male researchers. Author 1 is fluent in Polish, English, Arabic and Turkish and has lived in Syria for 1 year and Turkey for 2 years. Author 2 has lived in Turkey for 13 years, is employed as a professor at a Turkish university and is fluent in Turkish and English. Although ethnographic fieldwork unfolded over a period of a year, the depth of our “ethnographic knowing” (Pink and Morgan, 2013) emerged through layers of data collection and analysis that both preceded and expanded beyond our physical time in the field. We have both been researching Syrian migration to Turkey since 2016. We have also both volunteered with or led social responsibility projects for migrants.

We are Polish (Author 1) and American (Author 2) nationals, married to Muslim Turkish men. Thus, we are outsiders to both Turkish and also Syrian societies, but in some sense share the experience of being migrants to Turkey with our interlocutors. We are far more privileged than the women involved in the study and were perceived as such by the women. However, our long experience in Turkey and being perceived as “Turkish brides” facilitated some comfort and sense of understanding between us and the participants. Author 1 has two children which made it very easy for the participants to openly share information about parenting. In Istanbul, Author 2 was accompanied by an Arabic-speaking Syrian, Sunni Muslim research assistant. She was essential for securing migrants’ participation and making them feel comfortable during interviews as she knew many of them previously through a Turkish class and was herself a conservative, devout woman. (She has asked to remain anonymous, although we would have gladly attributed this work to her.).

This information about our backgrounds is important because of the chosen methodological framework, constructivist grounded theory, in which results stem from interactions between interviewers and interviewees (Charmaz, 2006). Thus, the positionality of the researchers is as important as the characteristics of the interviewees. The process of interviewing is seen as shaping the resulting data and directly connected to the relationship of trust that is created and in this sense is “constructed.” For this reason, a simple application of the concept of replicability is not possible (Anczyk et al., 2019). However, one way of verifying our data was achieved by the fact that we coded our data separately, then compared our coded materials and integrated them into more general categories that we developed (i.e., family, work, and neighborhood). This is similar to the procedure called “abbreviated grounded theory” by Willig (2008) in which another researcher cross-checks coded interview material.

### Sampling

Both of us used ethnographic fieldwork involving semi-structured interviews and participant observation. Author 2 drew on fieldwork carried out under a HORIZON 2020 project (name withheld to preserve anonymity). The study by Author 2 was reviewed and approved by Özyeğin University’s Ethical Review Board. For the research conducted in Hatay, Author 1 worked with ethical guidelines established by her advisor, Halina Grzymala-Moszczynska, as Jagiellonian University did not have an ethical review board at the time the research was conducted. All participants were provided oral and written information about the aim of the study and informed about confidentiality, benefits, risks and voluntary participation.

Author 1 was based in Iskenderun and Antakya, the two biggest cities of the Hatay province in South East Turkey, while Author 2 draws on research conducted in Istanbul. The central sampling criterion was to include women who had arrived within the last 10 years and whose presence was tied to humanitarian or asylum related provisions. Together, we conducted a total of 33 semi-structured interviews. Tables 1, 2 show the demographic characteristics of the participants, including age, regional origin in Syria, education, marital status, number of children, employment status and years having lived in Turkey. When participants are quoted throughout the article, we provide their age, marital status and number of children as a quick reference for readers (e.g., “30, married, 2” is the equivalent of 30 years old, married with 2 children). Our objective was to obtain a diverse sample with regard to location within our cities and to not only focus on refugees in NGO centers, living in city-centers or in “Syrian neighborhoods.” All interviewees were Sunni Muslims and Syrian nationals. There is variation in our sample with regard to the socio-economic background of migrants, but a majority were economically and socially precarious (Canefe, 2018).

**TABLE 1:** Demographic characteristics of participants (Study 1).

**Participant**	**Age (years)**	**Region of origin in Syria**	**Education**	**Martial Status**	**Children**	**Employment**	**Years in Turkey**
PN1	28	Saraqib/village	Primary	Married	2	No	3
PN2	43	Aleppo	Higher	Single	0	Office work	5
PN3	31	Aleppo	Secondary	In separation	1	No	4
PN4	30	Aleppo	Secondary	Married	3	No	6
PN5	42	Aleppo	Primary	Widow	1	Housekeeping/cleaning	4
PN6	53	Damascus	Secondary	Married	4	Hairdresser	5
PN7	29	Latakia	Secondary	Widow	2	Workhouse/factory	3
PN8	21	Hama/village	Primary	Single	0	Physical labor/farming	1,5
PN9	45	Hama/village	Primary	Married	5	Physical labor/farming	5
PN10	34	Hama/village	Primary	Married	2	Physical labor/farming	2
PN11	25	Hama/village	Primary	Married	2	Physical labor/farming	3
PN12	30	Aleppo	Primary	In separation	4	Housekeeping/cleaning	6
PN13	44	Idlib	Primary	Married	4	Physical labor/farming	4
PN14	46	Idlib	Primary	Married (disabled husband)	3	No	4
PN15	55	Aleppo	Primary	Married	5	No	4
PN16	27	Aleppo	Primary	Married	2	No	5
PN17	29	Aleppo	Primary	In separation	1	No	5
PN18	21	Damascus	University student	Single	No	Interpreter	5,5
PN19	35	Latakia	Higher	Single	No	NGO employee	7
PN20	27	Aleppo	Higher	Engaged	No	Interpreter	6
PN21	26	Aleppo	Primary	Married (2nd wife)	2	No	4

**TABLE 2:** Demographic characteristics of participants (Study 2).

**Participant**	**Age (years)**	**Region of origin in Syria**	**Education**	**Marital Status**	**Number of children**	**Employment status**	**Years in Turkey**
PN1	29	Damascus	Higher secondary	Married	0	Unemployed	3
PN2	45	Damascus	Higher secondary	Married	4	Unemployed	1
PN3	42	Damascus	Elementary school	Married	5	Unemployed	3
PN4	40	Damascus	Elementary school	Married	6	Unemployed	3
PN5	36	Aleppo	Elementary school	Married	6	Unemployed	3
PN6	46	Aleppo	Unknown	Married	3	Service employee or salesperson	3
PN7	40	Aleppo	Lower secondary	Divorced	5	Service employee or salesperson	1
PN8	34	Damascus	Higher secondary	Married	1	Specialist (teacher)	2
PN9	37	Damascus	Higher secondary	Married	2	Specialist (teacher)	5
PN10	26	Damascus	Higher secondary	Married	2	Unemployed	5
PN11	26	Damascus	Higher secondary	Married	2	Unemployed	4
PN12	27	Damascus	Higher secondary	Married	2	Unemployed	3

The recruiting process of interlocutors in both cities benefited from existing contacts between us and migrants, civil society actors and NGO members active in the field of refugee reception and integration. We established our first interviews via pre-existing contacts and gatekeepers and later combined this approach with snowballing. In Hatay, Author 1 has relatives through her husband who know migrants personally. They could thus facilitate introductions on the basis of neighborly relations, and being connected to the informants through such trusting relations was very important for obtaining their participation and willingness to discuss personal issues. In Istanbul, the contribution of the Syrian research assistant was significant for gaining participants’ trust and enabling open communication. Although the research assistant was not an anthropologist, she contributed many insights about women’s roles, goals and needs that enhanced the research.

Typically, research encounters took place in women’s homes and were preceded by small talk often including intermediary persons. We were introduced, the goals of the study were explained to participants and their consent was obtained. Before and after the interviews and observations in homes, we usually shared food and drink and had informal discussions with participants, sometimes for several hours. This approach contributed to establishing a trusting relationship. The interview guide initially covered (I) general question about the person, (II) their current everyday life in Turkey, including questions of housing, employment and educational activities, (III) their life in the country of origin, (IV) the migration journey, (V) their asylum procedure and status, and (VI) their current mental and physical health conditions. A variety of topics and themes were also added to the guide during the process of research as expected with a grounded theory approach. For example, when we noticed that the Islamic notion of “patience” was often mentioned, we added this as a topic to the guide and made it a focus of our coding. Each interview lasted approximately 1 h and was conducted in Arabic. The conversations were recorded, anonymized and transcribed into English.

In addition to interviews, we also conducted 6 months of participant observation in migrants’ homes, workplaces and in public spaces that migrants frequent, such as migrant neighborhoods or parks. Often other family members or neighbors were present. Observations of dress, spatial layout of apartments, furniture, and decorations were made. Photographs were also taken with participants’ consent. Most importantly, participant observation allowed us to observe interactions occurring naturally between migrants, co-nationals and locals and activities such as greeting, hosting, shared meals and more. We each took detailed field notes of participant observations, usually immediately after visits. However, the style of taking field notes being very personal (Emerson et al., 2011, pp. 15–17), we took notes in different forms. Further, some notes were more systematic, others journalistic in style or including more personal feelings and emotions, while still others were notes included in the transcripts of interviews. Therefore, it is difficult to quantify or even estimate the number of notes. At the end of the data collection process, each of us had 20–30 pages of notes.

### Data Analysis

We used a grounded theory approach, which involved identifying significant themes (initial coding) and then further coding of the interview transcripts and observations via focused coding (Charmaz, 2006). This form of constructivist grounded theory is appropriate because it has the flexibility to include unexplored categories and to present the richness and complexity of the female refugee lived experience, but it also possesses sufficient rigidness to ensure the academic quality of the work. First, in our initial coding, we looked at the manifest, descriptive dimension of the material, and how it could be condensed into meaning units. For example, we coded the challenges women describe, how they spend their time, and the role of religion in their lives. We then conducted a hermeneutic exploration of the latent, interpretive dimension of the material by reading and analyzing longer text passages for statements about agency. For example, the second level of focused coding enabled us to connect our initial coding of a woman’s difficulties like “my children are bullied” to a family-related stressor that demands a specific coping strategy from her. We applied “constant comparative methods” (Glaser and Strauss, 1967) at each level of analytic work to verify our theory development. In this form of grounded theory, “coding is the pivotal link between collecting data and developing an emergent theory to explain these data” (Charmaz, 2006, p. 46). Axial coding is not necessary as flexible guidelines are preferred (Charmaz, 2006, p. 61).

We each coded our own data independently and arrived at similar themes. Author 1 used MAXQDA and Author 2 used Nvivo (version 12) for software-supported content analysis of the interview material. This method allows for a systematic analysis of verbal communication and is apt for a contextualized reconstruction of personal perceptions (Krippendorf, 2004). We began comparing our results after interview codings were finalized, meaning that the possibility to influence one another in attributing meanings was limited. All of the translations for quotes in this article were done by us and the research assistant. All participants have been assigned pseudonyms to protect their identities.

## Results

We present our results in two sections. The first section focuses on the cultural context of stressors and the second section focuses on the cultural context of coping strategies. In each section, we further divide our findings into subsections. The section on stressors is focused on family, role- and place-related stressors. The section on coping examines faith-based coping, coping by rebuilding daily life routines and coping by rebuilding a sense of belonging.

### The Cultural Context of Stressors

The main stressors we observed are the result of social, cultural and economic factors and can be divided into three groups: family-related, role-related, and place-related.

#### Family-Related Stressors

Family-related stressors refers to situations or circumstances which are damaging to important others’ wellbeing, and, as such, they constitute a significant source of stress for a person. In most interviews, it was difficult for our participants to describe their individual problems. When we asked: “what are the biggest problems/difficulties you are facing in your life?”, they almost without exception answered by describing the problems of their children, parents, husband or other relatives. This recurring pattern of answers not matching the question (from the researcher’s perspective) may be explained by the fact that women define their personal wellbeing in terms of the wellbeing of their families. For example, one woman responded to this question by saying, “For me, the most difficult thing is that they bully my children in school. They call my son, “Syrian, Syrian, Syrian,” as if “Syrian” is an animal, not a human being. They call him names like “ISIS” or “dirty Syrian”” (29, widow, 2). Another woman related,

My biggest problem is that my daughters are sad that I can’t give them the things they want. Because you know, children don’t understand that we can’t afford it. I’m taking my daughter to the market and she wants this and that, but I can’t buy it. It’s sad because, before the war in Syria, we had everything we wanted (30, married, 3).

Through their children’s experiences, women evaluate their own worth and ability to mother, and they also confront what they personally have lost through the experience of migration.

Other common stressors in this category include discrimination of children at school, child labor, dropping out of school because of financial reasons and leaving elderly parents behind in Syria. One woman related tearfully that her mother in Syria is suffering from heart disease and does not have much longer to live. “My mother is ill, and I hoped to be with her and help her, but I can’t. We are trying to bring her here, but it is too hard” (42, married, 5). Such stressors relate to lost connections to wider kinship networks that contributed to a positive sense of self and security in the past.

Our participants’ statements show the importance of an interdependent sense of self (Kitayama and Uchida, 2005). From the perspective of individual agency, interdependent agency is often seen as passive, conformist, weak, irresponsible and ineffective. However, as our study shows, this form of agency also requires action, and it can be the most culturally sanctioned form available to Syrian refugee women. In addition to culture, gender and parental status also plays an important role in whether a person acts as an independent or interdependent agent, and agency needs to be evaluated in this light. We see the dichotomy between independent-interdependent agency as being part of a scale rather than two ends of mutually exclusive options. Although there may be cultural differences, any person surely can show independent and interdependent agency in different spheres of life.

#### Role-Related Stressors

Role-related stressors are circumstances or situation that causes stress by preventing people from fulfilling important family and social roles. This was an important problem described by participants, when they discussed being unable to properly perform motherhood duties (in their perception). One main cause was the necessity to work outside the home (mostly in hard, physical labor), which meant a painful change of social role for them, losing their femininity and the ability to be good mothers. This led to great distress, guilt and a feeling that their children’s childhoods are being disturbed. Several participants described how working affects their parenting:

When I first came from Syria, I used to leave my daughter with my sisters’ children. I had to work from 6 am to 6 pm. When I was coming back to pick her up, she was afraid of everything. She became aggressive. Then I found out that the other kids were beating her (42, widow, 1).

For those women who do not work, their children are their main priority. For example, Selma explained, “I am not doing anything for myself. I didn’t learn the language or complete my studies, because I always say, ‘my children are the priority now”’ (26, married, 2). We observed that women spend as much time as they can focusing on their children’s needs both material and also social, for example, teaching them Arabic by sending children to Qur’anic summer schools or Imam Hatip (religious) vocational schools to ensure cultural transmission. Other role-related stressors pertained to the inability of fulfilling daughter’s or sister’s duties, such as not being able to help a younger sister during pregnancy and labor.

#### Place-Related Stressors

The uprooting experience of forced migration has a special significance for those Syrian women who used to spend most of their time at home, which are the majority of women in our sample. Losing social connections to neighbors and relatives living close to home is an experience that affects every aspect of their lives, including parenting. For example, one woman explained, “In Syria, we were at home and our neighbors loved us, and we loved them back, but here. I have three kids, and they can’t make noise at home because we are afraid. Someone can always say that Syrians are noisy. We can’t get comfortable” (30, married, 3).

Neighbors’ discrimination is also a source of stress. For example, one woman related,

We do not communicate too much with the Turkish people because they don’t let us; my neighbors here, even when they are right in front of me, they don’t communicate. I tried to smile at one of them, but she turned her face away. They don’t want us. They refuse us. It is painful for me, imagine that they are my neighbors, and I cannot communicate with them! (45, married, 4).

Another woman related, “My Turkish neighbors are always gossiping, they say ‘look, Huda doesn’t have money.”’ We found that having friendly relations with neighbors and being invited for a coffee are given great importance during our interviews while the lack of neighborly relations is a serious stressor.

### The Cultural Context of Coping Strategies

Having laid out the main categories of stressors, we now turn to coping strategies, which we interpret as key manifestations of agency. We group these strategies into religious coping, coping by rebuilding home and family through daily life routines and coping by rebuilding belonging.

#### Religious Coping

When considering our participants’ coping, it is important to look closely at Islamic values, including patience, thankfulness, gratefulness and satisfaction, which they discuss repeatedly. These ideas emerged in conversation without prompting during our fieldwork or they were mentioned when a person was asked how she was getting along and what gives her meaning in life. Each are broad concepts that permeate Islamic worldviews and are also concretely referenced in Quranic verses (Achour et al., 2014). For example, *sabr*, was a key coping strategy for the majority of the participants. It is usually translated as patience or endurance in English, but does not correspond to the western idea of patience (understood as a passive waiting). Rather, it combines elements of acceptance, self-control and endurance (Achour et al., 2014). We were often told, “Islam means patience. Our religion is patience” (21, single, none). One woman related, “In the Islamic religion, patience is the most important thing. It’s the first rule. Our religion is patience. If God didn’t give us patience, we wouldn’t be able to carry on living” (34, married, 2). It is important to note that patience is seen as an active state of cooperating with God, as an effort expected from a believer. It was often discussed in the context of fate (*qadr*), seeing one’s situation in the context of God’s plan. *Sabr* means to actively live a moral life regardless of problems and perfect patience is to accept whatever comes from God. The old Arabic saying, “patience is a key to happiness,” was repeatedly explained to us during interviews.

The category of thankfulness or as most participants called it “saying *alhamdulillah*” for good and bad was present in almost every interview and is among most common coping strategies in everyday life. For example, one of the most powerful statements in our research came from an elderly woman, who reflected on the days just after her arrival to Turkey:

“When I first came from Syria I was crying and crying endlessly… but then I suddenly realized why did I come to this country? – Because God wants to see my patience. It is said: if our Lord loves us, he tests us to see our patience. He took my children and my mother away from me because he wanted to see my patience. So, then I thought: why am I crying? I shouldn’t cry – I should say *alhamdulillah*, I should rely on God. And so I organized Quranic meetings, and *zikr* (worship meetings), and frankly, I forgot everything that happened in Syria” (55, married, 5).

Interestingly, *sabr* (patience) and *shukr* (thankfulness) are also stages in spiritual development and part of the Sufi Path (Schimmel, 2011, p. 125). This is not to say that our participants were familiar with Sufi teachings as none of them mentioned that, but rather to draw attention to the fact that these categories are present in many layers of participants’ culture.

The need to be patient and grateful is something that members of the community encourage in one another. For example, when asked to describe relationships with relatives and friends, one woman explained,

Well, my family supports me a lot… They tell me to be patient, everything passes, thank God your kids have nothing wrong with them, they’re living, and come and see the situation here it’s a bit worse than yours. [cheerful tone] These are the things that make me forget my exhaustion after work and stuff (23, married, 2).

The role of mothers in the generational transmission of Syrian cultural values was also discussed by the participants. One participant stressed the importance of teaching gratefulness to her children:

My children come to me and say that other children at school have dads and money and a better life. I tell them – say *alhamdulillah*. I teach my children to say alhamdulillah for everything. Because if one knows to say *alhamdulillah*, he will not have any problems in life (29, widow, 2).

With such actions, mothers are also teaching children to be resilient and agentic (Lenette et al., 2013).

Satisfaction even in poverty and affliction are seen as a believers’ duty. We were told, for example, “God had ordained this fate upon us. We accept it and we are satisfied. This is something which comes from God. The Lord of the Worlds has ordained this, and his will is above everything” (21, single, none). Participants related that faith in god is a necessity.

Participants related that faith in god is a necessity.

Sometimes I feel sad and depressed, but I ask Allah for help a lot in my life. If I didn’t do that, I would have given up a very long time ago… so live your life as Allah chose for you and depend on Him. And, of course, better things are coming. It is said in the Quran that our future life is better than our current one. That doesn’t mean only our life after death, but also the life that you are living now (41, divorced, 5).

#### Coping by Rebuilding Home and Family Through Daily Life Routines

The second coping strategy we observed is related to rebuilding family life and creating a healthy atmosphere for children via trips, picnics, celebrations and other activities that transmit Syrian traditions. Such activities can be seen as a very effective coping strategy because they represent ways that women can do something to improve family life using very limited resources. For example, one woman related, “I try to make our lives in Turkey as normal as possible, to create a normal childhood for my children with childhood memories like the ones we had from Syria. I take them to picnics and to the seaside. We celebrate birthdays” (29, widow, 2). Another woman gave this example:

Every Friday or Saturday, when my kids are home, we make a cake or juice together. I make surprises for them for their birthdays, something simple that you can make at home, to make them happy. My daughter’s birthday is in few weeks so starting from now, I try to buy one ingredient for her birthday cake a week (30, married, 3).

Women participating in our research repeatedly stated that staying strong for their children and focusing on their needs contributed positively to their wellbeing and was a source of personal strength. For example, one woman explained,

I am a very strong woman. I am alone with my four children. I have to be strong for them because they take their strength from me. If I am weak, my children are also weak. Even if I am tired and unwell it’s enough for me to look at my children to feel better and stronger (30, separated, 4).

Another coping strategy mentioned by mothers was self-sacrifice, going without something for the sake of her children.

I have many problems here, but I can stand anything if my children are safe. My children give me strength. I would do anything for them, whatever they want. So sometimes I don’t eat, so I can buy milk for them. It makes me feel happy that I can give them something they want (44, married, 4).

While an outsider might interpret such actions as putting the woman’s needs below others’ needs and thus as a sign of her passivity and victimhood, understanding these women within their cultural context shows us that their children’s needs are fundamentally connected to their own well-being. It is also the only way they can fulfill their roles as mothers in a context of extreme poverty.

### Coping by Rebuilding Belonging

Neighborliness is an important cultural value and role for Syrian women and a way to enact proxy and collective agency. For example, Hoda explained how happy she was to know her neighbors, a process that took many years:

I started with learning the Turkish language, and when my language skills became better the situation became better, because you can join a conversation with Turkish people. My neighbors are old women, and when I come home, they like to tell me about their days; what they cooked, what were they feeling… and many other things (34, married, 1).

As losing social ties is an important stressor, rebuilding them is crucial for rebuilding one’s mental wellbeing. In the specific context of Syrian women in Turkey, whose mobility is restricted to the area of their residence for both economic and cultural reasons, often the only social ties they can have are the ones formed by neighbors. Having a neighbor who visits for coffee is not only a way of rebuilding the pre-war Syrian routine of everyday life. Being a neighbor and being able to invite neighbors for coffee or being able to be of help for them is important for a healthy sense of self. One woman related, “Meeting my neighbor is the thing that helps me the most. When I first met her and heard her speaking in an Aleppo dialect, it was like I saw Aleppo again. We spend a lot of time together” (30, separated, 4). Having good neighbors is so important that it even stops families from moving to a different part of the city, even if that would mean improving one’s housing conditions. For women who are accustomed to maintaining neighborly ties (Salamandra, 2004), a lack of social support is threatening to their coping process. Establishing strong relationships is an important resource as it increases opportunities for proxy and collective agency.

## Discussion: Cultural Coping for Studies of Migrant Women

Each of the inter-related stressors that migrant women face (family-related, role-related, and place-related) is deeply connected to an interdependent form of agency and women’s interactions with others (Kitayama and Uchida, 2005), to communion (Abele and Wojciszke, 2007) and to individual, proxy and collective agency (Bandura, 2008). It is important to perceive these stressors in their broader cultural and social context, because culture is one of the factors which creates and shapes them. For example, understanding what it means to lose a home in the fullest possible sense is necessary if we want to see the agency in parenting to the best of one’s ability, rebuilding a happy home and homeliness, and in trusting in god.

In much migration research, other stressors that refugees face (such as economic hardship, discrimination or political disenfranchisement) are not described in terms of how they affect the individual lived experience of the person concerned. We argue that it is only possible to understand migrants’ coping processes – and as a result to see their agency – if we see how such general stressors are “translated” by the culture and appraised by the individual. Based on the findings presented above, we will now examine three examples of such tailoring of the identified stressors (family-related, role-related, and place-related) according to the cultural context of Syrian women. Specifically, we look at the cultural impact on ideas of: mother’s role as stressor, work as stressor and losing home as stressor. Each of these are ways that general conditions of poverty and uprootedness are filtered through cultural constructs.

The association between mother’s psychological wellbeing and children’s adjustment following war is well documented in the psychological literature (Smith et al., 2001; El-Khani et al., 2017). Less well understood is the effect of cultural images of mothers’ roles and duties. In fact, it is stressors stemming from the cultural context, which are the most difficult aspects of deprivation for our participants and were topics discussed in detail in the majority of the interviews. Syrian women in our study view the world largely from an interdependent perspective, feeling the suffering of others as their own suffering and feeling responsible for others’ wellbeing. The inability to build a real childhood for their children, not introducing them to the Syrian curriculum and cultural heritage and not being home for them were commonly discussed. The change of a pattern of parenting to a more atomic one was also an important problem – usually the whole extended family was responsible for bringing up children in Syria. The loneliness and tiresomeness of the new reality of mothering in Turkey was an overwhelming stressor.

Limited time and lack of access to transportation are two stresses that affect women’s ability to fulfill their familial roles but may be overlooked during research and can even lead to misunderstandings and negative stereotypes about refugees’ passivity. Most refugee women live in poverty, but to fully comprehend the implications of the specific form of this deprivation one needs to listen very closely to the person who experiences it and observe her actions. For example, not having a washing machine or access to hot water does not at first seem like a serious stressor for a person coming from a warzone – but for a mother of two small children being forced to wash all clothing by hand leads to serious time poverty. And time poverty can be a main reason for not attending a Turkish course provided by authorities. This in turn can result in a negative stereotype of Syrian women as conservative or not wanting to learn or change their lives for the better. The amount of work put into washing clothes in such conditions escapes the attention of many researchers, as is often the case of invisible work of women. But in the specific context of keeping the house in exile, being clean and keeping children good-looking may be an important goal for a Syrian mother. Her ‘invisible’ effort, which is hardly ever a subject of research, works as bullying prevention at school and builds her own and her child’s self-respect. One of the mothers that we interviewed informed us that her neighbors are sometimes gossiping about her children being well-dressed despite being from a poor family, but she takes pride in mending, washing and ironing for them, and she says she does not lower her standards only because they are refugees. Thus, she enacts a very significant form of agency in presenting a well-dressed child to the world.

As we related, our informants complain about having to work because it keeps them from properly performing their mothering role. While a traditional feminist point of view might see refugee women working outside the home for the first time as a source of liberation and a sign of their agency, many Syrian women do not see it this way themselves. They are more likely to locate their agency in what they do for their children. It was often expressed by our participants that in Syria their life was better because they did not have to work outside the home. Being in public space was associated with getting tired (“it’s hot, and we are fasting, and we have to work for 8 h under the sun”) and feeling unsecure. The public space in Syria was often a place of sexual harassment of women and seen as unfit for women without a male relative present. However, the perception of work as yet another stressor also depends on the nature of the job. In most cases, our working participants were involved in physical labor, with bad employment conditions, so their work, while necessary for survival, could not contribute to improving their children’s situation. For those working in NGOs, work is perceived differently. Thus, an intersectional approach to agency is needed – all contextual factors need to be considered to understand what actions mean to migrant women.

Losing home for the participants of this study means not only losing a place of psychological safety and personal freedom, but also losing the connection to relatives and their local circle of female family members and neighbors, together with the possibility of mutual care and a feeling of connectivity and belongingness. Home is a complex concept, and as Rosbrook and Schweitzer (2010) put it “To lose one’s home is a pervasive loss, which is disorienting to the individual because it is complex, difficult to conceptualize and, some would argue, impossible to quantify.” This statement is even more valid in the context of Syrian culture which has been described as a culture of home and belongingness to place (Abi-Hashem, 2008). For these women, who experienced shattered social networks and were forced to spend even more time at home, staying in an “unhomely” space was a difficult experience. They liked to talk about and show photos of their houses in Syria, which they arranged after getting married. Some explicitly said that they do not invite anyone to their Turkish home, as they are ashamed of living in such a space. The loss of the neighborhood was especially disorienting as they were used to the close-knit communities of the “*hara*,” where women cooked and took care of children and elderly relatives together.

Just as the stressors we described are linked to interdependent senses of self, the coping strategies are likewise linked to important social relationships. Each category of coping strategy we identified comes from an intersection of religious, cultural and gender factors. Although we have organized religion into a separate category above (i.e., religious coping), and focus more on gender in the latter two categories (i.e., coping by rebuilding home and family through daily life routines and coping by rebuilding belonging), the interviews showed a strong overlap. Additionally, even religious categories such as “gratitude to God” can be used by people who do not consider themselves to be religious. Further, religious and ethnic or national categories may intersect. Some categories are derived directly from Quranic verses, but others are simply referred to as “we, Syrians” or “we, in Syria.” Some categories of coping are related to established gender roles – being a mother or a daughter or a neighbor brings different possibilities for coping with forced migration stressors – while other strategies may be less tied to gender roles and rather linked to cultural values.

Our study shows that religious coping is agentive. Existing case studies of refugee coping in the context of culture and religion support our finding that religion has a positive effect on forced migrants’ mental wellbeing (McMichael, 2002; Shoeb et al., 2007; Pahud et al., 2009; Darychuk and Jackson, 2015; El-Khani et al., 2017; Hasan et al., 2018; Shaw et al., 2019). The practical implications of religious coping and indigenous healing methods have been studied (Goździak, 2002), while research shows that elements of Arabo-Islamic culture, which overlap with the concepts presented in our findings support resilience and post-traumatic growth (Abi-Hashem, 2008; Punamäki, 2010). We find that behaviors such as religious reframing of the situation and being patient, hopeful and satisfied are the bedrock for undertaking any other actions to improve one’s condition.

Creating or recreating everyday routines for the family as well as organizing special events (such as, *iftar* holiday dinners and birthdays); cooking children’s favorite Syrian foods or helping him or her with their homework with Google Translate are efforts that can often be overlooked by researchers. Yet, they need to be seen as enactments of agency and as valuable investments in children’s’ resilience and well-being. These everyday actions are not just how women pass the time, but they actually make their lives meaningful.

A failure to consider the cultural context explains why analyses of the “sacrifices” made by refugee mothers for the sake of their children’s wellbeing is so often misunderstood. Such actions are often described as negative coping strategies – even if they are described by women themselves as having a positive influence on their motivation and psychological well-being. But if we acknowledge the moral injury caused by the inability to fulfill a mother’s role, we can understand the positive effect on their subjective wellbeing and also the agency they enact through this way of coping.

## Conclusion – Bringing a Culturally Contextual Theory of Women’s Agency to Migration Studies

Almost two decades ago Côté and Levine (2002, p. 10) called for psychologists and sociologists to combine their parallel understandings of agency in order to create a more integrated and comprehensive understanding of the term. They claimed that the main challenge lies in the fact that:

*“Sociology must acknowledge the notion that people are capable of “agentic” or intentional behaviors, even though they are often constrained by normed social structures (either subtly through socialization and enculturation or coercively through force).”*

This call stayed mostly unanswered, but the understanding and contextualization of agency progressed in each discipline separately, and the specific experiences of diverse social groups were gradually included within their corpuses of knowledge. The contributions of feminist theory and cultural psychology deepened our sensitivity to what agency can mean. The question we ask in this research is: how can we combine all of these insights into a theory of agency that is intersectional and flexible enough to observe agency in any configuration of circumstances? The need for an interdisciplinary discussion of a cultural and gender sensitive theory of agency is urgent, especially if we consider the possible negative effects of neglecting it whereby any perceived lack of agency (understood as efficacy) fosters negative stereotypes (Cuddy et al., 2008). By reviewing the stressors and coping strategies of Syrian women in Turkey, we have sought to demonstrate that this question can be answered by adopting a cultural coping model and taking culture and context into account. Refugee women’s agency is sometimes overlooked because it is shown in their private, everyday activities. However, these seemingly basic activities are crucial for rebuilding safe spaces and a satisfying life after being uprooted. Coping provides a valuable framework for reflecting on agency because it presumes that the goal is psychological health and well-being, not necessarily changing social structures.

Although it is important to describe refugee women’s everyday agency, this doesn’t mean that we need to take an overly celebratory approach, and thereby minimize the effect of oppressive social structures. Further, some of the attempts that women undertake fail and bring disappointment. It can also be argued that patience, satisfaction and other Islamic coping strategies keep Muslims from changing their situation. This is not only an “outsiders” opinion but did come up in one of our interviews. Nevertheless, these realities do not negate religious coping or mean that it is ineffective.

Having laid out a more comprehensive theory of agency, it is also important to note that some migrants enact a type of agency that we are more familiar with. For example, some women seem to be fighting patriarchy and redefining the meaning of war as a liberation from oppressive social systems. For example, one young woman whose husband left her without granting her a divorce (causing many serious problems for her) said:

I gave birth to my daughter here alone, and I’m struggling to bring her up here, and I’m teaching her everything, and I will not let any man or his family decide about her future […] that’s why I want to stay in Turkey, because here women’s rights are strong. In Syria women have no rights (29, separated, 1).

Such statements are a more traditional way of observing and describing agency. We do not object to such descriptions, but we argue that they should not be the only way of seeing refugee women’s agency. We can also see agency in their everyday attempts to mother and make new homes.

### Limitations

The qualitative nature of this study means that it relies heavily on self-reported data which brings certain limitations. For example, some events discussed by the participants (e.g., difficult experiences during their first months in Turkey) took place several years before the interview, meaning that reflections may have changed over time, and participants are communicating selective memories of these events. Also, some interviews took place in public spaces (i.e., schools and NGO facilities), which might have influenced the parts of the interviews where the local population’s attitudes toward Syrians were discussed. The sensitive nature of the questions asked during interviews required a certain level of trust between the interviewer and the interviewee. Because of the history of political and religious persecution in Syria, we cannot assume that this was always achieved. We noticed that participants were more willing to speak openly if the interviews took place in their homes and were preceded by an introductory meeting with known contacts. This was possible for the majority of our interviews, but not all of them. Finally, because of the chosen methodology, the replicability of this study might be limited to secondary coding of the transcribed interviews, rather than the collecting of interviews from a different set of participants.

Despite its limitations, our research strongly suggests that studies of agency would benefit from identifying stressors and coping strategies according to the specific cultural context. By attending to cultural contexts, migration researchers can escape the trap of viewing women as passive victims, and they may find more effective ways to support them in their everyday struggles. For mental health practitioners working with refugee populations, it is important to understand the culturally-grounded stressors and coping strategies.

## Data Availability Statement

The datasets presented in this article are not readily available because the data generally contains sensitive information about a vulnerable group and thus, it should not be shared openly. However, interested parties may contact SR about access to anonymized RESPOND Project data. Requests to access the datasets should be directed to susan.rottmann@ozyegin.edu.tr.

## Ethics Statement

The studies involving human participants were reviewed and approved by Özyeğin University - ERB (part of RESPOND project) for research in Istanbul. For the research conducted in Hatay, ethical review and approval was not required for the study on human participants in accordance with the local legislation and institutional requirements. In Hatay the researcher worked with ethical guidelines established by her advisor, Halina Grzymala-Moszczyńska and followed the rules of ethical research described in The Routledge Handbook of Research Methods in the Study of Religion (Bird et al., 2011). Written informed consent for participation was not required for this study in accordance with the national legislation and the institutional requirements.

## Author Contributions

All authors listed have made a substantial, direct and intellectual contribution to the work, and approved it for publication.

## Conflict of Interest

The authors declare that the research was conducted in the absence of any commercial or financial relationships that could be construed as a potential conflict of interest.

## Publisher’s Note

All claims expressed in this article are solely those of the authors and do not necessarily represent those of their affiliated organizations, or those of the publisher, the editors and the reviewers. Any product that may be evaluated in this article, or claim that may be made by its manufacturer, is not guaranteed or endorsed by the publisher.
